# Targeting PD-1/PD-L1 pathway in myelodysplastic syndromes and acute myeloid leukemia

**DOI:** 10.1186/s40164-022-00263-4

**Published:** 2022-03-02

**Authors:** Xingcheng Yang, Ling Ma, Xiaoying Zhang, Liang Huang, Jia Wei

**Affiliations:** 1 Department of Hematology, Tongji Hospital, Tongji Medical College, Huazhong University of Science and Technology, 1095 Jiefang Avenue, Wuhan, 430030 Hubei China; 2 Immunotherapy Research Center for Hematologic Diseases of Hubei Province, Wuhan, 430030 Hubei China; 3 Department of Clinical Laboratory, Union Hospital, Tongji Medical College, Huazhong University of Science and Technology, Wuhan, 430022 China

**Keywords:** Myelodysplastic syndrome, Acute myeloid leukemia, Programmed cell death-1, Programmed cell death ligand-1, Immune checkpoint, Hypomethylating agent, AML transformation

## Abstract

Myelodysplastic syndromes (MDS) and acute myeloid leukemia (AML) are clonal hematopoietic stem cell diseases arising from the bone marrow (BM), and approximately 30% of MDS eventually progress to AML, associated with increasingly aggressive neoplastic hematopoietic clones and poor survival. Dysregulated immune microenvironment has been recognized as a key pathogenic driver of MDS and AML, causing high rate of intramedullary apoptosis in lower-risk MDS to immunosuppression in higher-risk MDS and AML. Immune checkpoint molecules, including programmed cell death-1 (PD-1) and programmed cell death ligand-1 (PD-L1), play important roles in oncogenesis by maintaining an immunosuppressive tumor microenvironment. Recently, both molecules have been examined in MDS and AML. Abnormal inflammatory signaling, genetic and/or epigenetic alterations, interactions between cells, and treatment of patients all have been involved in dysregulating PD-1/PD-L1 signaling in these two diseases. Furthermore, with the PD-1/PD-L1 pathway activated in immune microenvironment, the milieu of BM shift to immunosuppressive, contributing to a clonal evolution of blasts. Nevertheless, numerous preclinical studies have suggested a potential response of patients to PD-1/PD-L1 blocker. Current clinical trials employing these drugs in MDS and AML have reported mixed clinical responses. In this paper, we focus on the recent preclinical advances of the PD-1/PD-L1 signaling in MDS and AML, and available and ongoing outcomes of PD-1/PD-L1 inhibitor in patients. We also discuss the novel PD-1/PD-L1 blocker-based immunotherapeutic strategies and challenges, including identifying reliable biomarkers, determining settings, and exploring optimal combination therapies.

## Introduction

Myelodysplastic syndromes (MDS) are a heterogeneous group of clonal myeloid neoplasms originating from hematopoietic stem cells (HSCs). MDS has a potential high risk of developing to acute myeloid leukemia (AML), which has been an increasing global burden during the past 28 years [1, 2]. About 30% of MDS cases eventually transform into AML, which is diagnosed by a blast amount of ≥ 20% of total nucleated cells in BM [3]. Recurrent genetic mutations, epigenetic changes and aberrant immune signaling pathways have been implicated in disease progression, contributing in a multistep and evolutionary way to MDS/AML transformation [4–7]. On one hand, genetic and epigenetic mutations in blasts confer these mutant cells with capabilities of self-renewal and clonal progression [6]. On the other hand, the inflammatory milieu in the BM of patients imposes selective pressure both on mutant clones and on normal hematopoietic stem/progenitor cells (HSPCs), further driving the emergence of subclones, which exhibit a competitive advantage in the milieu over normal HSPCs [8, 9]. In addition, recent studies also have shown a connection between epigenetic alterations and dysregulated inflammatory pathways, including EZH2 and KDM6B signalings that play important roles in leukemic transformation, was implicated in the dysregulated innate immune responses observed in MDS/AML patients [10–16]. In general, lower-risk MDS is often linked to increased intramedullary apoptosis and pyroptosis, accompanied by elevated interferon-γ (IFN-γ), tumor necrosis factor-α (TNF-α), interleukin-6 (IL-6), S100A8/S100A9 and NLRP3 inflammasome levels [17–19]. While in higher-risk MDS and AML, the milieu in BM is relatively more immunosuppressive, as effector T cells and NKs are exhausted and functionally impaired, accompanied by elevated frequencies of hyperfunctional regulatory T cells (Tregs) [20–26]. Furthermore, immune checkpoint molecules, including PD-1, cytotoxic T-lymphocyte-associated-protein 4 (CTLA4), T-cell immunoglobulin mucin-3 (TIM-3), T cell immunoglobulin and ITIM domain (TIGIT), lymphocyte activation gene-3 (LAG-3), also have critical functions in this process by protecting blasts from host immune surveillance [27–32]. Mounting evidence has demonstrated the dynamic function of PD-1/PD-L1 signaling in promoting leukemogenesis in MDS/AML, thus increasing attention are drawn to this field.

Physiologically, PD-1 is expressed on activated T cells, Tregs and B cells. PD-1 prevents immune overactivation by binding to its receptor PD-L1/PD-L2 found mainly on macrophages and dendritic cells (DCs) [33–37]. Following binding to PD-L1, the intracellular tyrosine residue of PD-1 is phosphorylated, followed by subsequent SHP-1 and SHP-2 recruitment, which disrupts a series of downstream molecules in TCR signaling, e.g., PI3K/AKT, RAS-ERK1/2 and PKCδ signaling. This in turn promotes effector T cells apoptosis and inhibits proliferation and cytokine secretion of these cells [38–42]. However, this protective function of the PD-1/PD-L1 signaling can also be utilized by tumors to maintain an immunosuppressive tumor microenvironment, favoring cancer cell proliferation [43]. In general, PD-1 binding to PD-L1 favors tumor evasion mainly via the following mechanisms: (1) inhibition of effector T cell function by promoting apoptosis and decreasing proliferation of T cells, and blocking the production of cytokines; (2) downregulation of T-cell receptor (TCR) expression and induction of proliferation in Tregs, which are key mediators suppressing cytokine secretion and proliferation of effector T cells [44–47]; (3) protection of tumor cells by reversely transmitting anti-apoptotic signals through the PD-L1 receptor; (4) formation of a ‘molecular shield’ to induce resistance against T cell–mediated killing, including Fas- and staurosporine-mediated apoptosis, leukemia-specific cytotoxic T lymphocyte-induced cytolysis and interfered signal transducer and activator of transcription 3 (STAT3)/caspase-7 dependent interferon-mediated cytotoxicity [48, 49]. In addition, reverse signaling mediated by PD-L1 further promotes tumor growth by enhancing glycolytic metabolism in PD-L1^+^ tumor cells via the Akt/mTOR pathway [50, 51]. In several preclinical studies of AML established in murine models, PD-1/PD-L1 signaling was implicated in leukemia development, e.g., inhibition of host antitumor immune responses, exhaustion of CD8^+^ T cells and promotion of Treg-mediated effector T cell suppression [52–54].

By removing inhibitory effects of PD-1/PD-L1 pathway, PD-1/PD-L1 blocker enhance antitumor immune responses and exhibit optimal therapeutic efficacy in various tumor types, including melanoma [55], non-small-cell lung cancer [56] and renal-cell cancer [57]. Notably, upregulated PD-1 levels in tumor-infiltrating lymphocytes and PD-L1 levels in tumour cells constitute reliable biomarkers for predicting responses in individuals administered with PD-1/PD-L1 blocker [58–61]. Recently, upregulated PD-1/PD-L1 levels has also been found in patients with myeloid malignancies, including MDS and AML, and both cell culture and animal experiments have strongly suggested potential benefits of PD-1/PD-L1 blocker in preventing progression of these disease [31, 54, 62–66]. However, although numerous preclinical studies have suggested promising efficacy in preclinical MDS/AML models, current clinical outcomes of PD-1/PD-L1 blocker in MDS/AML remains controversial. Exploring roles of PD-1/PD-L1 signaling in BM microenvironment and ameliorating patients’ responses to PD-1/PD-L1 blocker deserves urgent attention.

In the current review, we mainly focus on PD-1/PD-L1 dysregulation in MDS/AML, including the following aspects: (1) the pattern of aberrant PD-1/PD-L1 expression in MDS/AML; (2) the mechanisms by which dysregulated PD-1/PD-L1 signaling influences the BM microenvironment; (3) the mechanisms by which abnormal inflammatory signaling, genetic and/or epigenetic alterations and interactions between cells regulate PD-1/PD-L1 expression; (4) the mechanisms by which drugs, including hypomethylating agents (HMAs), affect PD-1 expression in MDS/AML. Finally, the available outcomes of completed and ongoing clinical trials on PD-1/PD-L1 blocker are reviewed. These studies have examined the therapeutic applications of such molecules either as single or combined agents in MDS/AML.

### Dysregulated PD-1/PD-L1 pathway in MDS/AML pathogenesis

#### Pattern of aberrant PD-1 and PD-L1 expression in MDS/AML

In the past decades, PD-L1 and PD-1 expression in patients’ immune microenvironment have been well investigated. Recent studies have demonstrated that these markers are aberrantly expressed in MDS and AML patients. Detailed results noted that PD-L1 was mainly upregulated in CD34^+^ blasts, while PD-1 was increased in effectors T cells and Tregs [23, 25, 26, 30, 31, 62, 63, 66–79]. Table 1 summarizes current studies focusing on the dysregulated PD-L1 and PD-1 axis in MDS and AML as assessed by flow cytometry.

**Table 1:** Dysregulated PD-L1 and PD-1 expression in MDS/AML

Disease	Upregulated molecules	Reference	Specimen types and numbers	Cell subtypes with upregulation of PD-1/PD-L1	Clinical outcome associations
MDS	PD-L1	Cheng et al. [62]	BM (n = 10)	CD34^+^ cells; CD33^+^ CD14^+^ MDSCs; CD71^+^ erythroid progenitors	NA
		Kondo et al. [31]	BM (n = 40)	CD34^+^ cells	Higher PD-L1 expression was correlated with higher risk IPSS category
		Tcvetkovet al. [67]	BM (n = 57)	CD34^+^ cells	Higher PD-L1 expression was mostly seen during maturation of myeloid blasts into granulocytes
		Yang et al. [68]	BM (n = 69)	CD34^+^ cells	Higher PD-L1 expression was correlated with disease subtypes (MDS 2008 WHO classification) and a trend towards worse survival
		Montes et al. [69]	PB (n = 69)	CD34^+^ cells	Higher PD-L1 expression was seen in MDS rather than sAML
		Moskorz et al. [70]	BM (n = 7)	CD34^+^ cells	Higher PD-L1 expression was seen in CD38^+^ subset compared to CD38^−^ subset
		Sallman et al. [80]	BM (n = 107)	CD34^+^ cells	PD-L1 expression on HSCs was not correlated with blast percentage in BM, disease progression or IPSS-R risk categories
	PD-1	Cheng et al. [62]	BM(n = 10)	CD4^+^ /CD8^+^ T cells; CD34^+^ HSPCs; CD71^+^ erythroid progenitors	NA
		Yang et al. [68]	PB (n = 24)	PBMNCs	Higher PD-1 expression was correlated with older age
		Meng et al. [30]	PB(n = 26)	CD4^+^ /CD8^+^ T cells	Higher PD-1 expression was correlated with higher risk IPSS category
		Coats et al. [26]	PB (n = 26)	CD4^+^ effector memory cells; CD4^+^ memory cells; CD4^+^ TNF-α secreting cells; CD4^+^ /CD8^+^ naive T cells; Tregs	PD-1 expression was not correlated with disease stages
		Tcvetkov et al. [67]	BM (n = 57)	CD4^+^ /CD8^+^ T cells	NA
AML	PD-L1	Brodská, et al. [71]	PB(n = 36)	CD45dimSSC gating blasts	Higher PD-L1 expression was correlated with poor OS in patients with concomitant FLT3-ITD and NPM1 mutations
		Tamura et al. [72]	BM (n = 36)	CD34^+^ cells	NA
		Dong et al. [66]	BM (n = 65)	CD45dimSSC gating blasts	NA
		Zhang et al. [73]	BM (n = 79)	CD34^+^ cells	Higher PD-L1 expression was observed in AML-M5 according to FAB classification, and correlated with a higher relapse rate
		Berthon et al. [63]	BM (n = 79)	CD34^+^ cells	Levels of PD-1 expression was not correlated with NPM1 or FLT3 mutations in newly diagnosed patients;
		Krönig et al. [74]	BM (n = 154)	CD34^+^ cells	Levels of PD-L1 expression was not correlated with blasts load
		Williams et al. [75]	BM (n = 107)	CD34^+^ cells	Higher PD-L1 expression were observed in patients harboring TP53‐mutation and complex cytogenetics
		Wu et al. [76]	PB (n = 22)	Vδ2 T cells	NA
	PD-1	Wan et al. [25]	PB (n = 45)	Tregs	NA
		Tang et al. [77]	PB (n = 50)	CD4^+^ /CD8^+^ /γδ T cells	Higher PD-1 expression on CD8^+^ T cells was correlated with poor OS and EFS
		Dong et al. [66]	BM (n = 65)	Tregs	Higher PD-1 expression on Tregs was correlated with poor OS and DFS, and suggested a trend of higher frequencies PD-L1^+^ blasts in BM
		Daver et al. [78]	BM(n = 74)	CD4^+^ /CD8^+^ / Tregs	NA
		Williams et al. [75]	BM (n = 107)	CD4^+^ /CD8^+^	NA
		Schnorfeil et al. [79]	PB (n = 37); BM (n = 44)	CD4^+^ /CD8^+^ T cells	Levels of PD-1 expression was not correlated with CMV serostatus
		Tan et al. [23]	PB (n = 30); BM (n = 15)	CD3^+^ /CD8^+^	NA

*BM* bone marrow, *EFS* event-free survival, *ITD* internal tandem duplications, *PBMNCs* peripheral blood mononuclear cells, *MDSCs* myeloid-derived suppressor cells, *NA* not applicable, *NPM1* nucleophosmin, *sAML* secondary acute myeloid leukemia, *Tregs* regulatory T cells

Identifying specific associations between PD-1/PD-L1 levels and disease status is necessary. Clear description of these aspects could aid the determination of the most suitable time for drug administration in the specific subgroup of patients who are more likely to show good responses to PD-1/PD-L1 inhibition.

In MDS, Kondo et al. indicated that expression of PD-L1 was only observed in individuals with 5% or more blasts, and found a correlation between higher PD-L1 levels and high-risk International Prognostic Scoring System (IPSS) categories [31]. However, some studies reported contradictory findings. Montes et al. observed that PD-L1 levels on peripheral blood (PB) CD34^+^ cells were comparable between different disease stages of MDS and even higher in MDS than in secondary AML (sAML) [69]. Additionally, Sallman et al. analyzed PD-L1 levels on bone marrow mononuclear cells by flow cytometry, and found no association between PD-L1 levels on HSCs, blast percentage in BM, disease progression and Revised International Prognostic Scoring System (IPSS-R) risk categories [80]. Furthermore, although several studies have confirmed upregulated PD-1 on T cells in MDS by flow cytometry [26, 30, 62, 67, 68], it remains unknown whether PD-1 levels are associated with disease progression. Meng et al. detected higher PD-1 level on PB effector T cells in the higher-risk IPSS group [30]. Moreover, Coats et al. analyzed PB PD-1 levels on several T-cell subgroups, e.g., CD4^+^/CD8^+^ naive T cells, CD4^+^ TNF-α cells, CD4^+^ memory cells, CD4^+^ effector memory cells and Tregs. However, the latter study identified no marked level differences for these markers between the low- and high-risk IPSS-R groups [26].

In AML, most studies [63, 72, 74] but not all [66] found the frequency of PD-L1^+^ CD34^+^ cells are comparable between newly diagnosed patients and healthy donors. While in relapsed/refractory (R/R) cases and CR patients with IFN-γ exposure, PD-L1 levels were confirmed to be significantly increased [63, 66, 72, 74]. Further research suggested specific FAB subtype, age, complex cytogenetics and some somatic mutations had significant associations with elevated PD-L1 expression on CD34^+^ blasts. Zhang et al. found that the AML-M5 subtype showed higher PD-L1 expression than the others [73]. Brodská et al. observed that FLT3 and NPM1 were associated with PD-L1 upregulation [71, 73]; meanwhile, Williams et al. found that AML blasts with TP53 mutation were more frequently positive for PD‐L1, which was further confirmed by the studies on MDS and sAML patients by Sallman et al. and Zeidan et al. [75, 80, 81]. However, no correlation of PD-L1 expression with blast load was noted [63, 73, 80]. Notably, researchers also revealed high PD-L1 expression on both PB and BM leukemia blasts upon diagnosis was correlated with poor prognosis [29, 51, 71].

As effector T cells are essential in inducing anticancer immunity, exhausted state of T cells in AML has attracted substantial interest. In BM samples, PD-1 levels on CD4^+^/8^+^ T cells and Tregs [78] were found to be significantly increased in newly diagnosed cases and even higher in relapsed AML cases compared with healthy donors (HD), or only restricted on CD4^+^ T cells in relapsed AML [75]. In PB specimens, studies found PD-1 expressions on CD4^+^, CD8^+^ [75], Vδ2^+^ T cells [77] and Tregs [66] were upregulated in AML patients upon diagnosed, while others argued that PD-1 upregulation on CD8^+^ T cells [79] were only limited to relapsed cases.

The relationship between frequencies of PD-1^+^ T cells and survival have also been analyzed. Dong et al. found that elevated PD-1^+^ Treg rate predicted poor overall survival (OS) and disease-free survival (DFS) [66]. Tang et al. reported that increased percentage of PD-1^+^ CD8^+^ T cells in AML patients, both upon diagnosis and after induction chemotherapy, were linked to poor OS and EFS [77].

Several hypotheses have been formulated to explain these discrepant results. Firstly, as MDS and AML are heterogeneous and genetically unstable, the disease state among patients may be genetically different even for the same risk category according to IPSS-R or European LeukemiaNet (ELN) [82, 83]. Secondly, variable treatments received by patients prior to their assessment could influence PD-1 and PD-L1 expressions [68, 84]. Finally, different types of specimens may also lead to the incomparability of results [85]. Additional studies with larger sample sizes are required to obtain definitive results.

#### Roles of aberrant PD-1/PD-L1 signaling in MDS/AML pathogenesis

**Fig. 1:**
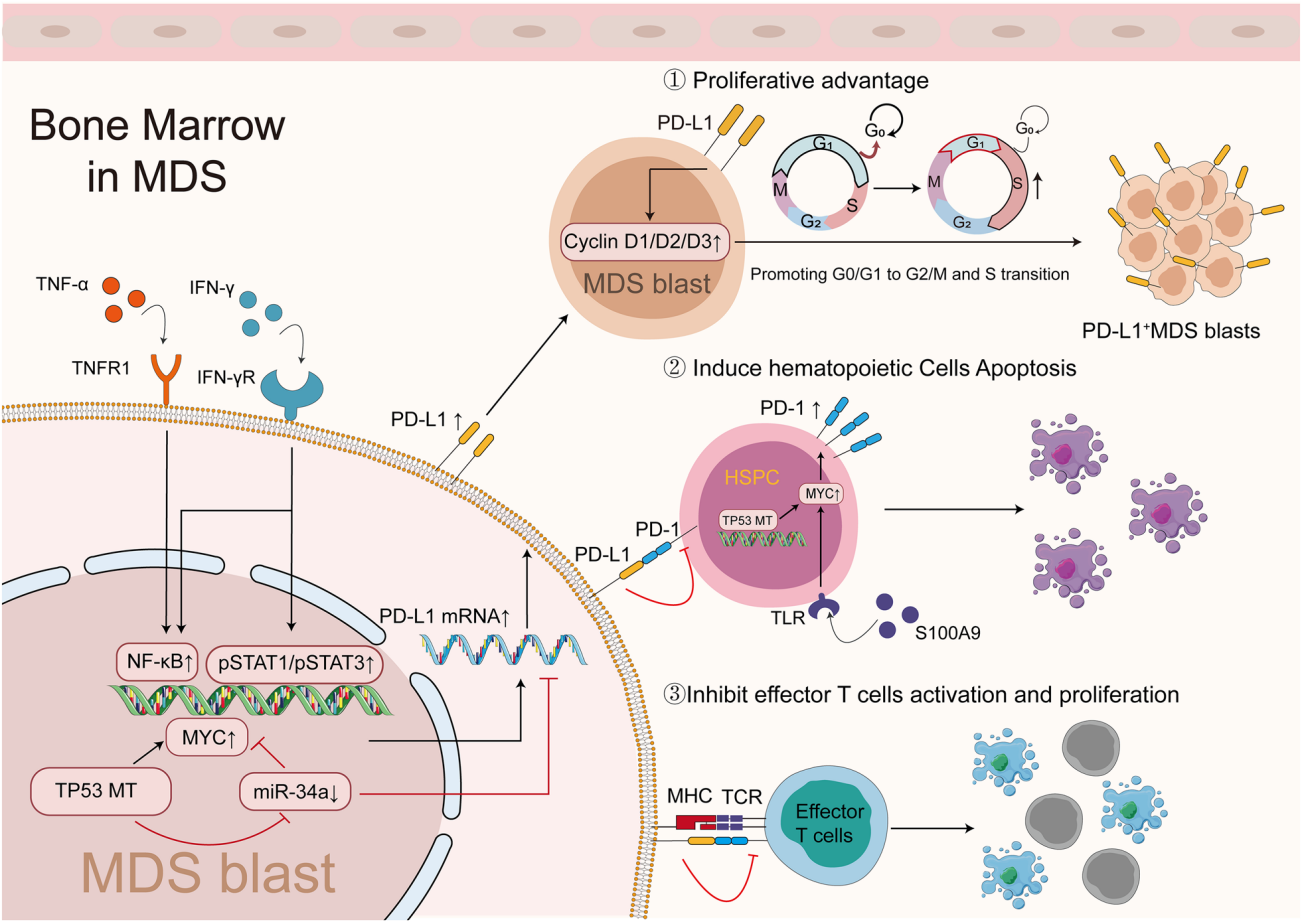
Function of dysregulated PD-1/PD-L1 pathway in MDS. Upon exposure to IFN-γ and TNF-α, PD-L1 levels are increased in MDS blasts via NF-κB and pSTAT1/pSTAT3 activation. Further, TP53 mutation also implicated in PD-L1 upregulation via MYC upregulation and miR-34a downregulation, thus regulating PD-L1 levels at a post-transcriptional level. In CD34^+^ HSPCs, TP53 mutation and S100A9 upregulate PD-1 via MYC. Furthermore, PD-L1^+^ MDS blasts mediate pathogenesis through PD-1/PD-L1 signaling, by the following mechanisms: ① blasts expressing PD-L1 confer proliferative advantages, expressing higher levels of CyclinD1/D2/D3 and growing more actively; ② the binding of PD-L1 on MDS blasts with PD-1 on CD34^+^ HSPCs result in PD-1^+^CD34^+^ HSPC apoptosis. ③ the binding of PD-L1 on MDS blasts with PD-1 on CD4^+^/CD8^+^ T cells inhibit the activation and proliferation of these effector T cells. MHC, major histocompatibility complex; TNFR, TNF receptor; MT, mutation; pSTAT, phosphorylated signal transducer and activator of transcription

**Fig. 2:**
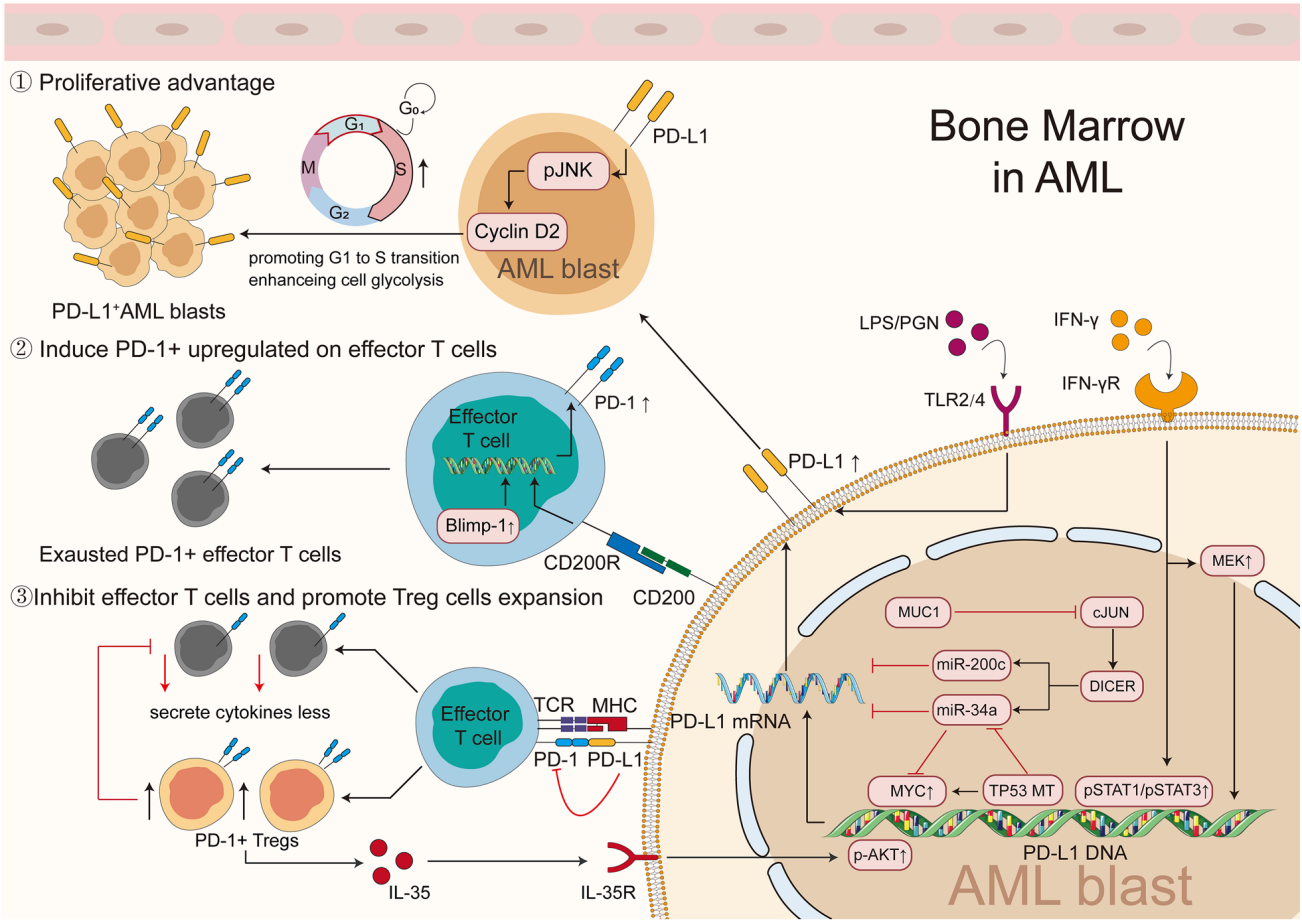
Function of dysregulated PD-1/PD-L1 pathway in AML. Upon exposure to IFN-γ and TNF-α, PD-L1 levels are increased in MDS blasts via MEK and pSTAT1/pSTAT3 activation. Similar to MDS blasts, TP53 mutation also plays important roles in PD-L1 upregulation via MYC upregulation and miR-34a downregulation, thus regulating PD-L1 levels at a post-transcriptional level. In addition, miR-34a and miR200c are regulated by DICER, cJUN and MUC1. Furthermore, PD-L1^+^ AML blast-mediated pathogenesis occurs through PD-1/PD-L1 signaling, by the following mechanisms: ① blasts expressing PD-L1 confer proliferative advantages, including enhanced cell glycolysis and higher levels of Cyclin D2, via activation of pJNK, resulting in more active growth; ② the interaction of CD200 on AML blasts with CD200R on effectors leads to the upregulation of PD-1, which is also regulated by increased Bmilp-1, promoting the inaction of these effector T cells; ③ the binding of PD-L1 on AML blasts with PD-1 on effector T cells suppress activation of these effector T cells, and promote conversion of Tregs from conventional CD4^+^ T cells, which triggers the secretion of IL-35 and upregulates PD-L1 on AML blasts via pAkt activation

In preclinical studies, several roles for PD-1/PD-L1 signaling in MDS/AML have been identified. These evidence offers hypotheses on using PD-1/PD-L1 blocker to suppress disease progression. Several mechanisms underpinning PD-1/PD-L1 signaling dysregulation have been implicated in the pathophysiology of leukemogenesis, including conferring PD-L1^+^ CD34^+^ blasts a proliferative advantage, hematopoietic cell apoptosis and immune evasion. Figures 1 and 2 depict these possible mechanisms.

##### Proliferative advantage

The growth of HSCs is controlled in a delicate cell cycle to ensure successful cell division and maintain tissue homeostasis, which is dysregulated in malignant clones [86]. In addition to the known roles of PD-L1 in immune suppression via interaction with PD-1, recent findings have demonstrated that PD-L1 can promote leukemogenesis by activating its own downstream signaling pathway. Kondo et al. observed that PD-L1^+^ blasts isolated from MDS patients exhibited a growth advantage compared with PD-L1^−^ blasts. In addition, PD-L1^+^ cell rate was lower at the static stage (G0/G1 phase) and higher at the synthesis stage (S and G2/M phases) [31]. Further studies demonstrated that these PD-L1^+^ cells incorporated more BrdU and expressed higher Ki-67 level. Consistently, in vitro experiments revealed that increased cyclin D1, D2 and D3 mRNA amounts in PD-L1^+^ cell populations compared with PD-L1^−^ cell populations derived from SKM-1 and F-36P MDS cell lines. Higher numbers of PD-L1^+^ colonies were also found in the colony-forming assay [31]. Similar results were obtained in the AML murine model, and Fang et al. noted that PD-L1 on blasts was critical in promoting leukemogenesis in vivo via CD274/JNK/Cyclin D2 signaling. PD-L1 deletion resulted in significant decrease in leukemia-initiating cells (LICs) count and caused G1 phase arrest. Meanwhile, in PD-L1^−^ cells, there was significantly decreased phosphorylation of JNK/Cyclin D2, which were key regulators in promoting G1-S transition during the cell cycle [87]. Moreover, Ma et al. reported that higher PD-L1 levels on MOLM-13 cells were correlated with higher glycolysis‑associated genes expressions, including ALDOA, PGK1, LDHA and HK2, and a higher level of glycolysis was also observed. After transfection with PD-L1-sh1/2, these cells showed significantly high apoptosis rates [51].

##### Hematopoietic cell apoptosis

Ineffective hematopoiesis is one of the hallmarks of MDS, leading to blood cytopenias [1]. It was reported that dysregulated PD-1/PD-L1 signaling was responsible for this process [62]. In the bone marrow milieu of MDS, increased PD-L1 and PD-1 levels in CD34^+^ cells as well as increased PD-1 levels in CD71^+^ erythroid progenitors were observed. Cheng et al. further demonstrated that coculture of PD-1^+^ CD71^+^ erythroid progenitors and PD-1^+^CD34^+^ HSPCs with the addition of recombinant human PD-L1 resulted in significantly increased amount of activated caspase-3 in these PD-1^+^ cells. This induced cell death and ineffective hematopoiesis, but could be reversed by further administration of PD-1/PD-L1 blocker [62].

##### Immunosuppression

The immunosuppressive effect of PD-1/PD-L1 signaling was also notable in leukemogenesis of MDS and AML. Studies showed that PD-L1^+^ blasts from MDS/AML patients had a higher resistance to effector T cell-mediated killing, which was partially abolished by PD-L1 blockade [31, 63, 64]. In addition, PD-L1 produced by blasts could promoted the conversion of Tregs from conventional CD4^+^ T cells in AML, and upregulated PD-1 on these Tregs [66, 88]. Such Tregs secreted more IL-10 and IL-35, which not only could inhibit effector T cell function, but also were capable of inducing chemoresistance of HL-60 cells to cytarabine, and promoting proliferation of these cells [66]. Zhou and colleagues noted that PD-1/PD-L1 interaction was critical in Treg-induced immunosuppression in vivo. The latter study showed that Tregs from PD-1 KO mice were less capable of dampening the function of WT CD8^+^ T cells. And the proliferation and function CD8^+^ T cells were significantly rescued after PD-L1 blockade, followed by the eradication of established AML in the murine model [54].

Nevertheless, Schnorfeil et al. found that in a part of AML cases relapsing after chemotherapy or allo-SCT despite of PD-1 upregulation on both PB CD4^+^ and CD8^+^ T cells, proliferation and cytokine production in these PD-1^+^ effector T cells remained functionally intact [79].

Accordingly, the above findings indicate that PD-1/PD-L1 signaling is critical in promoting leukemogenesis, and further adding PD-1/PD-L1 blocker augment immune response in effector T cells and induce apoptosis in MDS/AML blasts.

#### Regulation of PD-1/PD-L1 signaling in MDS/AML

As mentioned above, dysregulated PD-1/PD-L1 signaling has vital functions in the BM milieu of MDS and AML. Therefore, it is critical to investigate the mechanisms of PD-1/PD-L1 signaling regulation in MDS and AML. Recent studies have revealed that many factors are implicated in this process, e.g., abnormal inflammatory signaling, genetic mutations or epigenetic alterations and cell–cell interactions.

##### Inflammatory signaling

Proinflammatory cytokines have long been recognized to affect the pathogeneses of MDS and AML [16, 62, 89–91]. IFN-γ, TNF-α, S100A9, PGN and LPS [19, 63, 92], are dysregulated in patients (especially higher in the lower-risk MDS group) [93–96] and could strongly induce PD-L1 upregulation, suggesting a role for PD-L1 in modulating the immune microenvironment. Kondo et al. indicated that treatment of SKM-1 cells, F-36P cells or MDS blasts with IFN-γ and TNF-α elevated PD-L1 mRNA and protein levels. Additional studies indicated that inhibition of NF-κB could block PD-L1 upregulation mediated by IFN-γ and TNF-α [31], suggesting a vital role for NF-κB signaling in regulating PD-L1 expression. S100A9, which is produced by myeloid-derived suppressor cells (MDSCs) and mediates premature death of HSPCs, exhibits a variable expression pattern at different disease stages of MDS [16, 17, 97]. Cheng et al. found that S100A9 was also implicated in PD-1/PD-L1 upregulation. After exposure to S100A9, PD-1 in CD34^+^ HSPCs and CD71^+^ erythroid progenitor cells and PD-L1 on MDSCs were upregulated; further studies indicated MYC activation upon S100A9 exposure, resulting in upregulations of these two molecules [62].

Studies on AML have found that IFN-γ can induce PD-L1 levels on blasts through multiple mechanisms. STAT1/STAT3 and MAPK pathways were implicated, providing variable targets to suppress tumor tolerance and induce immunity against AML [63, 65, 98, 99]. Yoyen-Ermis et al. found that pSTAT3 was upregulated in blasts isolated from MDS/AML patients and THP-1 cell lines after IFN-γ exposure; further investigation showed that Stattic, a small-molecule inhibitor of STAT3/STAT1, was efficient in blocking IFN-γ-induced PD-L1 upregulation [65]. By injecting CpG-*Stat3* siRNA into mice AML cells, Hossain et al. confirmed that STAT3 was implicated in PD-L1 regulation, and could induce AML cell immunogenicity by upregulating the proportions of CD8^+^ T cells in vivo [98]. Additionally, the MAPK pathway attributed a role to regulate PD-L1 expression. Studies found that after expose to IFN-γ, blasts from AML patients and murine models showed increased PD-L1 expression, which was blocked by MEK inhibitor [63, 99].

##### Genetic or epigenetic alterations

Inflammatory signaling is not the only stimulus linked to PD-1 and PD-L1 dysregulation in MDS/AML. In addition to extrinsic cellular factors, TP53 alteration was also reported to impact PD-L1 expression [80]. Sallman et al. demonstrated that TP53-mutant HSCs derived from MDS/AML expressed more PD-L1 with concomitant overexpression of MYC and downregulation of miR-34a. The latter played a major role in MYC degradation [80]. Notably, in the latter study, although HSCs and hematopoietic progenitor cells (HPCs) expressed CD34 and harbored TP53 mutations, increased PD-L1 expression was largely restricted to HSCs, with no significant difference in HPCs, suggesting that a more accurate classification of CD34^+^ cells for evaluating PD-L1 expression was required. Furthermore, miR-34a played a role in suppressing PD-L1 expression by binding to the 3′UTR region of PD-L1 mRNA [100]. Pyzer et al. found that silencing of MUC1 in THP-1 and MOLM-14 cells caused significant PD-L1 downregulation [101]. Further studies demonstrated that the underlying mechanism was mediated by c-Jun activity suppression, which in turn downregulated the microRNA-processing protein DICER and ultimately upregulated miR-200c and miR-34a, which negatively regulated PD-L1 levels in AML, as also noted by other studies [62, 80, 101]. In addition, epigenetic alterations also play an essential role in regulating PD-1/PD-L1 expression in MDS/AML. Previous studies reported that following a prolonged TCR stimulation in CD8^+^ T cells, promoter of PD-1 was demethylated, which further upregulated PD-1 level [102]. Recently, HMAs have been shown to exert epigenetic immunomodulatory and demethylation effects on tumor cells [103]. Their capacity to upregulate PD-1 on effector T cells in some MDS/AML patients was also noted [68, 84]. Further studies focusing on the mechanisms by which HMAs influence PD-1 expression are warranted.

##### Cell interactions

In AML, PD-1 expression on effector T cells is modulated at the protein level by interactions between cells. CD200, overexpressed in AML blasts with a suppressive role in the antitumor response [104], was also recently observed to be linked to PD-1 expression (105). Coles et al. indicated that CD8 T cells from CD200hi AML patients showed higher levels of PD-1, almost twice, compared with CD200lo patients. Further in vitro assays suggested that CD200-CD200R interactions could lead to a significant PD-1 level upregulation on CD8^+^ T cells [106].

DCs were also found to be implicated in PD-1 expression regulation [107]. Lecciso et al. examined a cohort of AML patients and found that PD-1^+^ Tregs were significantly increased after combined daunorubicin and cytarabine chemotherapy. Further in vitro investigation revealed that ATP was critically involved in PD-1 upregulation, which was released from AML cells after daunorubicin treatment. When DCs were treated with ATP or cocultured with daunorubicin-treated AML cells, indoleamine 2,3-dioxygenase 1 (IDO1) was upregulated in DCs, which could induce PD-1^+^ Tregs. Notably, in vivo studies showed that in ATP receptor-lacking mice, daunorubicin failed to induce IDO1-expressing DCs and Tregs generation [107].

#### Association of dysregulated PD-1 and PD-L1 expression with HMA resistance

HMAs, including azacitidine (AZA) and decitabine (DAC), are the most common first-line treatment options for higher-risk MDS patients and older/unfit AML patients. However, approximately 50% of patients showed responses to these agents, with usually a transient duration [108]. Patients who lost responses to HMAs exhibited particularly poor survival, with an estimated median OS of 4.3 ~ 5.6 months [109, 110]. The mechanisms of resistance to HMAs in the setting of MDS have been actively explored. Diverse molecular mechanisms contributing to HMA resistance have been proposed, including integrin α5-mediated hematopoietic progenitor cell quiescence [111], elevated CDA/DCK ratio [112], increased RNA:m5C and NSUN1-/BRD4-associated active chromatin [113], elevated BCL2L10 expression (114), disturbed STAT3/5 signaling [115] and high number of mutations in the DNA methylation pathway, notably in TET2 gene [116–119].

Recent studies have identified a correlation between HMA resistance and dysregulated PD-1/PD-L1 signaling. Both PD-1 and PD-L1 levels indicated an increased trend in peripheral blood mononuclear cells (PBMNCs) of MDS/AML patients with no response to HMAs [68]. Notably, although baseline PD-1 expression was comparable between responders and non-responders, methylation in PD-1 promoter was markedly enhanced in the latter group, which had a more dynamic change of demethylation in the PD-1 promoter region and higher PD-1 protein expression (68). Furthermore, since PBMNCs were composed of different cell subpopulations in addition to T cells, Orskov et al. demonstrated that baseline PD-1 promoter methylation in effector T cells was higher in non-responders. In addition, individuals with higher demethylation levels in the PD-1 promoter exhibited a shorter OS, in accordance with poor survival noted in HMA-failure patients [84]. Furthermore, Yang et al. modeled the data in vitro and demonstrated that HMAs induced the upregulation of PD-L1 in KG-1 and THP-1 cells [68]. Upregulation of NF-κB and increased IFN-γ sensitivity were suggested to be responsible for this effect [120, 121].

**Fig. 3:**
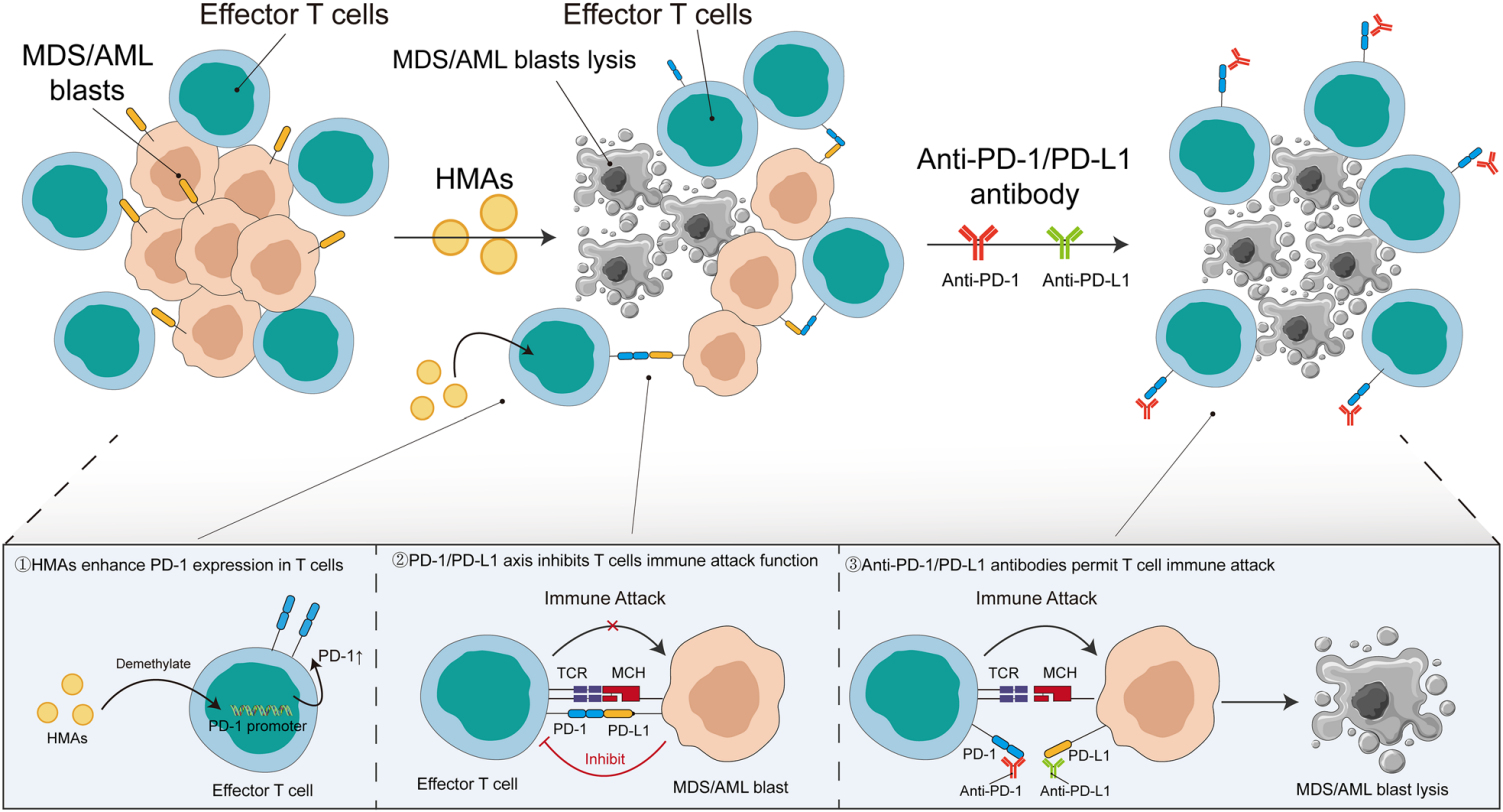
Immune microenvironment of the BM in HMA-failed MDS/AML patients. Following HMA therapy, a portion of MDS/AML blasts died, while other MDS/AML blasts survived and acquired HMA resistance, which can be further eradicated by PD-1 or PD-L1 inhibitors. The underlying mechanisms are as follows: ① following HMA therapy, PD-1 promoter methylation in CD8^+^ T cells is decreased, resulting in PD-1 upregulation; ② the activation of CD8^+^ T cells is suppressed by the binding of PD-1 expressed on CD8^+^ T cells and PD-L1 expressed on MDS/AML blasts; ③ further administration with PD-1/PD-L1 antibodies prevents the interaction of these two molecules, alleviating the activation of CD8^+^ T cells, which induces apoptosis in the remaining MDS/AML blasts

Figure 3 summarizes the immune microenvironment of the BM in HMA-failure MDS/AML patients. Given that resistance to HMAs may be mediated by increased PD-1/PD-L1 signaling, combining PD-1/PD-L1 blocker and HMAs was suggested for the treatment of the patients.

### Available clinical outcomes of PD-1/PD-L1 blockade-based therapy in MDS/AML

Based on preclinical findings that demonstrate PD-1 and PD-L1 are upregulated in MDS and R/R AML, especially in patients developing resistance to HMAs, the efficacy of several monoclonal antibodies targeting this pathway as single agent or combination therapies in treatment-naïve and HMA-failure MDS or R/R AML patients has been assessed. All available clinical results are summarized in Table 2.

**Table 2:** Available results of clinical trials for PD-1/PD-L1 inhibitor in treatment of MDS/AML

Reference	Trial phase	Patient characteristics	Intervention	Target	Efficacy	Toxicity
Berger et al. [122]	I, single-arm	8 R/R AML (4 AML relapsed after allo-SCT) and 1 MDS	CT-011 (0.2–6 mg/kg)	PD-1	One AML patient achieved peripheral blasts reduction	50% AML patients experienced grade 3–4 AEs and died (due to fulminate resistant leukemia but not study drug)
Garcia-Manero et al. [127]	Ib, single-arm	28 HMA-failure MDS	Pembrolizumab 10 mg/kg, Q2weeks	PD-1	ORR 4%; CR 0% OS rate 49% at 24 weeks	Hypothyroidism (14%), fatigue (11%), 7% grade 3/4 treatment-related AEs: 1 gastroenteritis (grade 3) and 1 TLS (grade 4)
Garcia-Manero et al. [128]	II, multi-arms non-randomized	15 HMA-failure MDS	Nivolumab 3 mg/kg on day 1 and 15 Q4weeks	PD-1	ORR 35%; CR/CRp 15%; mOS NR	Skin rash (11%); fatigue (9%); pain (7%); infection (6%); FN (5%); pruritus (6%); diarrhea (5%); constipation (4%); nausea (4%); ALT elevations (3%); anorexia (3%); and cough (3%) One early mortality
		20 HMA-failure MDS	Ipilimumab 3 mg/kg Q3weeks	CTLA-4	ORR 13%; CR/CRp 0%; mOS 8 mos	
		20 treatment-naïve MDS	Nivolumab 3 mg/kg on day 6 and 20, plus AZA 75 mg/m^2^ daily for 7 days Q4weeks	PD-1 + HMA	ORR 75%; CR/CRp 50%; mOS 12 mos	
		21 treatment-naïve MDS	Ipilimumab 3 mg/kg on day 6, plus AZA 75 mg/m^2^ daily for 7 days Q4weeks	CTLA-4 + HMA	ORR 71%; CR/CRp 38%; mOS 8 mos	
Chien et al. [132]	II, single arm	17 treatment-naïve MDS	Pembrolizumab 200 mg Q3weeks, plus AZA 75 mg/m^2^ daily for 7 days Q4weeks	PD-1 + HMA	ORR 76%; CR 3%; mOS NR	Arthralgias (40%), pneumonia (33%), nausea (27%). One patient died within first 60 days due to unrelated cause of ventricular fibrillation
		20 HMA-failure MDS	Pembrolizumab 200 mg Q3weeks, plus AZA 75 mg/m^2^ daily for 7 days Q4weeks	PD-1 + HMA	ORR 25%; CR 5%; mOS 5.8 mos	Pneumonia (32%), arthralgias (24%), constipation (24%). Two patients died within first 60 days
Daver et al. [131]	II, single arm	70 R/R AML (25 HMA-naïve and 45 HMA-failure)	Nivolumab 3 mg/kg on day 1 and day 14, plus AZA 75 mg/m^2^ daily for 7 days, Q4-6 weeks	PD- 1 + HMA	ORR 33% (58% in HMA-naïve,22% in HMA-failure patients); CR/CRi was 22%; mOS 6.3 mos	23% grade > 2 immune toxicities, 9 pneumonitis, 6 nephritis, 3 immune related skin rash, and 2 transaminitis
Gerds, et al. [129]	Ib, multi-arm non-randomized	10 HMA-failure MDS	Atezolizumab 1200 mg Q3weeks	PD-L1	ORR 0%; CR 0%; mOS 5.9 mos	10% grade > 3 FN; 0% died (10% occurred within 3 months)
		11 HMA-failure MDS	Atezolizumab 1200 mg Q3weeks plus AZA 75 mg/m^2^ daily for 7 days Q4weeks	PD-L1 + HMA	ORR 9%; CR 0%; mOS 10.7 mos	36% grade > 3 FN; 64% died (18% occurred within 3 months)
		21 treatment-naïve MDS	Atezolizumab 840 mg Q2weeks plus AZA 75 mg/m^2^ daily for 7 days Q4weeks	PD-L1 + HMA	ORR 62%; CRp 14%; mOS NR	33% grade > 3 FN; 29% died (all occurred within 3 months)
Zeidan, et al. [134]	II, multi-arms randomized controlled	42 treatment-naïve MDS	Durvalumab 1500 mg Q4weeks plus AZA 75 mg/m^2^ daily for 7 days Q4weeks	PD-L1 + HMA	ORR 61.9%; CR 7.1%; mOS 11.6 mos	Most common treatment-emergent AEs were hematologic and gastrointestinal toxicity. Immune-mediated AEs were observed in 7 MDS and 17 AML patients
		42 treatment-naïve MDS	AZA 75 mg/m^2^ daily for 7 days Q4weeks	HMA	ORR 47.6%; CR 9.5%; mOS 16.7 mos;	
		64 treatment-naïve AML 1–7 every 4 weeks	Durvalumab 1500 mg Q4weeks plus AZA 75 mg/m^2^ daily for 7 days Q4weeks	PD-L1 + HMA	ORR 31.3%; CR 17.2%; mOS 13.0 mos	
		65 treatment-naïve AML	AZA 75 mg/m^2^ daily for 7 days Q4weeks	HMA	ORR 35.4%; CR 21.5%; mOS 14.4 mos	
Zeidner et al. [136]	II, single arm	37 R/R AML	HiDAC 1.5/2 gm/m^2^ daily for 5 days plus Pembrolizumab 200 mg on day 14. Responders were continued to receive pembrolizumab 200 mg Q3weeks	PD-1 + chemotherapy	ORR 46%; CR 38%; mOS 8.9 mos	Most frequent grade > 3 pembrolizumab-related toxicities were ALT elevation (n = 1), AST elevation (n = 1), and grade > 3 maculopapular rash (n = 2). One patient did not survive due to disease progression within sixty days
Ravandi et al. [137]	II, single arm	44 treatment-naïve (42 AML and 2 high-risk MDS)	cytarabine 1.5 g/m^2^ daily for 4 days and idarubicin 12 mg/m^2^ daily for 3 days, plus Nivolumab 3 mg/kg on day 24 ± 2 and continued Q2weeks	PD-1 + chemotherapy	ORR 80%; CR 78%; mOS 18.54 mos	6 patients had seven grade 3/4 IRAE with rash (n = 2), colitis (n = 2), transaminitis (n = 1), pancreatitis (n = 1) and cholecystitis (n = 1)

*AE* adverse events, *AZA* 5-azacytidine, *CR* complete response, *CRp* CR with incomplete platelet recovery, *FN* febrile neutropenia, *HiDAC* high dose cytarabine, *IRAE* immune-related adverse events, *mOS* median overall survival, *NR* not reached, *ORR* overall response rate, *SAE* serious adverse event, *TLS* tumor lysis syndrome

#### PD-1/PD-L1 inhibitor as monotherapy-based agents

Pidilizumab was the first PD-1 inhibitor examined in AML/MDS, and 8 AML patients (in both frontline and R/R settings) and one MDS patient were included in one phase I trial. Pidilizumab was relatively safe, showing no dose-limiting or treatment-related adverse events. As shown in this trial, only one AML patient showed minimal response, exhibiting a decreased peripheral blast number from 50 to 5%. No trial employing pidilizumab has been conducted in MDS/AML subsequently [122].

Other PD-1/PD-L1 inhibitor examined in MDS include pembrolizumab, nivolumab and atezolizumab, which have shown encouraging clinical outcomes such as long-term survival in solid tumors [123–126]. In MDS, pembrolizumab monotherapy was examined in a phase Ib study (NCT01953692), which enrolled 28 MDS patients following failed responses to hypomethylating agents [127]. A total of 5.6-month median follow-up was conducted, and the overall response rate (ORR) was only 4%, with an OS rate of 49% at 24-week. The efficacy of nivolumab was also evaluated in MDS patients following HMA failure in a phase II trial, which used this antibody as a monotherapy-based agent (NCT02397720) [128]. No CR was detected, and only two patients achieved partial response (PR) (13% ORR) among 15 patients, and the median OS was 8 months. Although the efficacy of PD-1 antibodies used as monotherapy was unimpressive in HMA-failure cohorts, their safety profile was generally favorable in these trials and no treatment-related deaths were reported [128]. However, a phase Ib trial on atezolizumab, the only PD-L1 antibody that was examined as a monotherapy agent in MDS patients following HMA failure, reported 70% (7/10) of patients did not survive after a 160-day median follow-up, mainly due to disease progression [129].

Although preclinical studies suggested potential benefits of PD-1/PD-L1 blocker for MDS/AML, especially after HMA failure, current clinical outcomes after applying such agents as monotherapy were disappointing. The causes of this discrepancy between preclinical studies and clinical trials are under investigation. The dynamic changes in the immune microenvironment of the bone marrow may be critical, as previous studies have reported that some individuals with promoter-demethylated PD-1 gene during HMA therapy develop a diverse remethylation pattern in the same loci before the next treatment cycle [84]. Other potential explanations of this discrepancy may be that the majority of available trials enrolled high-risk patients with a heavy disease burden and advanced tumor stage, which also reflects a poor prognosis [130].

#### Combination therapy of PD-1/PD-L1 inhibitor in MDS/AML

Although preclinical investigations suggested potential synergistic effects of HMAs or intensive chemotherapy and PD-1/PD-L1 inhibitor, current clinical trials reported mixed responses, with data mostly unimpressive in HMA-failure and R/R patients.

In a phase II study including 70 R/R AML patients (25 HMA-naïve and 45 HMA-failure) treated with AZA and nivolumab, the ORR was 33% (58% and 22% for HMA-naïve and HMA-failure, respectively), the CR/CRi rate was 22%, and the median OS was 6.3 months [131]. The authors reported that the outcomes were better in this trial than those described in a historical cohort study performed in the same institution with an ORR of 20% after HMA-based salvage therapy for 172 R/R AML patients [131]. Notably, after 2 and 4 doses of nivolumab, CTLA-4 levels were markedly increased on CD4^+^ T cells in non-responders [131].

Another phase II trial (NCT03094637) assessed the synergistic effects of pembrolizumab and AZA in MDS. Totally, 37 MDS patients (17 HMA-naïve and 20 HMA-failure) with IPSS intermediate-1 or higher-risk disease were enrolled. The ORRs were 76% in HMA-naïve cohort and 25% in the HMA-failure cohort, with a CR of 18% and 5%, respectively [132]. Notably, one HMA-failure patient who harbored 2 separate TP53 gene mutations experienced stable disease with transfusion independence for more than 34 months in this trial, and ASXL1 and SETBP1 gene mutations were most frequently found in responders of the HMA-failure cohort, while TET2, ASXL1 SRSF2 and RUNX1 gene mutations were detected in the HMA-naïve cohort [132, 133]. Garcia-Manero et al. reported the outcomes after administering nivolumab with AZA in a phase II trial [128]. The trial enrolled 20 treatment-naïve MDS patients, and found an ORR of 75% and a CR/CRp of 50%. The median OS was 12 months [128]. Still, the safety profile of PD-1 blocker in combination with HMAs was favorable in these trials. While in the phase Ib trial employed atezolizumab combined with AZA (NCT02508870) [129] in 32 high-risk MDS patients (21 HMA-naïve and 11 HMA-failure), although a relatively high response was reported in the HMA-naïve cohort with an ORR of 62% and a CR of 14%, an unexpected early mortality was noted. Six patients (29%) did not survive within 3 months after therapy initiation, owing to serious treatment-related adverse events (AEs) [129]. In the HMA-failure cohort, a modest response was reported with an ORR of 9%, no CR, and a median OS of 10.7 months. Totally, 7 patients (64%) did not survive because of disease progression, with a median survival time of 299 days [129]. Unexpected early mortality rate led to early termination of this trial.

Currently, only one randomized trial conducted on PD-1/PD-L1 inhibition therapy for treatment-naïve MDS/AML patients, and reported similar outcomes compared to the HMA-naïve cohorts in the above-mentioned trial [134]. In a phase II trial (NCT02775903), a total of 84 higher-risk MDS and 129 older AML (aged ≥ 65 years) patients were enrolled. Patients were randomly grouped to receive either durvalumab in combination with AZA or AZA alone. The ORR was merely improved in the PD-L1 inhibitor combination group versus the AZA monotherapy group (61.9% and 31.3% in the MDS and AML cohorts in the combination group, respectively, vs. 47.6% and 35.4% in the MDS and AML cohorts in the AZA alone group, respectively), whereas no improvement was found in median OS (11.6 and 13.0 months in MDS and AML patients in the combination group, respectively, vs. 16.7 and 14.4 months in MDS and AML patients in the AZA alone group) [134]. In this trial, importantly, although PD-L1 gene promoter demethylation was confirmed in patients following AZA administration, PD-L1 protein expression upregulation in blasts was not observed [134].

In addition to HMAs, intensive chemotherapy was also suggested to potentially exert synergistic effects with PD-1/PD-L1 blocker [107, 135]. In a phase II trial, 37 R/R AML patients were administered with high dose cytarabine in combination with pembrolizumab, and ORR was 46% and CR rate was 38%, with an 8.9 months median OS. AEs including febrile neutropenia (FN) (57%), ALT elevation (43%) and AST elevation (32%) were most commonly seen. One patient did not survive due to disease progression within 60 days [136]. In another single-arm phase II trial, 44 treatment-naïve patients (42 AML and 2 high-risk MDS) were treated with cytarabine, idarubicin and nivolumab; the ORR was 80%, and 78% patients achieved CR [137]. Median OS was 18.54 months, with no significant improvement compared to a contemporary cohort examining cytarabine plus idarubicin [137, 138]. Notably, analysis conducted in this trial showed that non-responders had markedly elevated percentage of bone marrow CD4^+^ T cells co-expressing PD-1/TIM-3 in comparison with responders [137].

Accordingly, the above data suggested PD-1/PD-L1 blocker combined with HMAs or chemotherapeutics could yield more promising results in treatment-naïve patients, whereas they could also increased the frequency of AEs compared with single agents. Although the patients administered with combination therapy showed more promising responses, only a modest improvement was seen compared with historical controls. The factors accounting for these discrepancies between preclinical studies and clinical trials are not completely understood. Reasonable explanations may include differences between disease models and patients, as the immune microenvironment in patients is more complex with various groups of immune cells and mesenchymal cells. It should also be noted that MDS and AML are a group of highly heterogeneous diseases, and genetic subtypes are usually different among patients, even in the same risk group. Patients who carry specific somatic mutations, including TP53, ASXL1, SETBP1, TET2, SRSF2 and RUNX1 gene mutations, are more likely to respond to PD-1/PD-L1 inhibitor [133, 139]. Next-generation sequencing (NGS) is rapidly changing the clinical decision-making process in MDS/AML [140–144]. The use of NGS and its combination with further molecular data may yield a high predictive power. Furthermore, double or more immune checkpoint inhibitor (ICI)-based combination therapies may be more efficient. Finally, most of the trials mentioned above are non-randomized studies with inadequate sample size, and more comparative and randomized trials with larger sample size across different patient populations and longer follow-up periods are required in the future.

#### Ongoing trials exploring PD-1/PD-L1 blockade-based strategies

Since current trials have reported limited efficacy of PD-1/PD-L1 blocker in MDS/AML, numerous ongoing efforts are being made for assessing the effectiveness of novel combinations, including with novel HMAs, other immune checkpoint inhibitors (ICIs), histone deacetylase inhibitors (HDACi), tumor vaccines or chemotherapeutic agents (Table 3). In general, several challenges are under investigation. Firstly, in NCT03092674, a randomized II/III phase study with approximately 1,670 participants, the efficacy of nivolumab alone or in combination with AZA for a large MDS patient population was examined. Secondly, in addition to patients in first-line or relapsed /refractory setting, PD-1/PD-L1 blockade are also being examined for effectiveness in MDS/AML relapsing after allogeneic hematopoietic stem cell transplantation (allo-HSCT) or individuals at high risk of relapse in NCT03286114, NCT02981914, NCT02532231, NCT03600155, NCT02846376 and NCT02771197. It should be noted that although previous reports have demonstrated increased efficacy of PD-1/PD-L1 blocker in MDS/AML cases after allo-HSCT, unfavorable high risk of graft-versus-host disease (GVHD) was also reported [145–147]. The optimal dose of nivolumab for alleviating graft-versus-leukemia (GVL) without severe GVHD induction required to be defined. Thirdly, researchers are also interested in exploring optimal managements of HMA in combination with PD-1/PD-L1 inhibitor, including the optimal dose, administration time and delivery methods (NCT03969446 and NCT02281084), as well as other novel HMAs (NCT02935361 and NCT02892318). Furthermore, PD-1/PD-L1 inhibitor are also examined in combination with other immunotherapeutic agents, including LAG-3 inhibitor, (NCT04913922), CTLA-4 inhibitor (NCT02530463), TIM-3 inhibitor (NCT03066648), CD47 inhibitor (NCT03922477), CD-33 and OX-40 inhibitor (NCT03390296), tumor vaccines (NCT03358719) or chemotherapy (NCT04541277, NCT04214249, NCT04722952 and ChiCTR2100045296). Finally, combinations of PD-1 inhibitor with HDACi are also being studied (NCT02936752, NCT04277442 and NCT04284787).

**Table 3:** Ongoing clinical trials for immune checkpoint inhibitors in treatment of MDS/AML

Phase	Inclusion	Therapy	Target	Primary objectives	Reference
Ib	Relapsed MDS/AML/ALL after allo-SCT	Pembrolizumab	PD-1	CR, PR, SD, toxicity	NCT03286114
Early I	Relapsed MDS/AML/Hodgkin and non-Hodgkin lymphoma after allo-SCT	Pembrolizumab	PD-1	AEs	NCT02981914
II	High risk for relapse AML	Nivolumab	PD-1	RFS	NCT02532231
Ib	Relapsed MDS/AML after allo-SCT	Nivolumab and/or lpilimumab	PD-1 and/or CTAL-4	MTD, DLTs, toxicity	NCT03600155
I	High risk for relapsed MDS/AML after allo-SCT	Nivolumab and/or lpilimumab	PD-1 and/or CTAL-4	Safety	NCT02846376
II	Non-favorable risk AML	Pembrolizumab + Flu/Mel + Allo-SCT	PD-1 + Lymphodepletion + Allo-SCT	RFS	NCT02771197
I	Previously untreated and relapsed/refractory MDS/AML	Pembrolizumab + DAC	PD-1 + HMA	AEs, MTD, CR/CRi	NCT03969446
II/III	Previously untreated higher-risk MDS	Nivolumab + AZA/AZA	PD-1 + HMA	OS	NCT03092674
II	R/R AML	Camrelizumab (SHR-1210) + DAC	PD-1 + HMA	ORR, CR	NCT04353479
0	Previously untreated higher-risk MDS	Sintilimab + DAC	PD-1 + HMA	ORR	ChiCTR2100044393
II	Higher-risk MDS	Camrelizumab + DAC	PD-1 + HMA	ORR	ChiCTR1900028440
IV	MDS-EB1/2	PD-1 monoclonal antibody + AZA	PD-1 + HMA	Efficacy	ChiCTR2000034927
IV	HMAs failure MDS	Camrelizumab + DAC	PD-1 + HMA	ORR	ChiCTR2100044210
II	previously untreated AML/sAML	Pembrolizumab + Ara-C + IDA/DNR	PD-1 + chemotherapy	MDR-CR	NCT04214249
II	R/R AML excluding relapsed after HSCT	Tislelizumab + CAG	PD-1 + chemotherapy	ORR	NCT04541277
–	Higher-risk MDS	Tislelizumab + HMA + cytarabine	PD-1 + HMA + chemotherapy	Efficacy	ChiCTR2100045296
III	R/R AML	Visilizumab + Azacytidine + HAG regimen	PD-1 + HMA + chemotherapy	CR, CRi, PR	NCT04722952
II	R/R AML	Nivolumab + AZA + Relatlimab	PD-1 + LAG-3	MTD, DLTs, ORR	NCT04913922
II	Previously untreated or HMAs failure MDS	Nivolumab; nivolumab + lpilimumab; nivolumab + lpilimumab + AZA	PD-1 and/or CTAL-4 and/or HMA	ORR	NCT02530463
I	AML and intermediate or high- risk MDS	PDR001 + DAC and/or MBG453	PD-1 + TIM-3 and/or HMA	AEs, DLTs	NCT03066648
Ib/II	R/R AML	Avelumab + AZA + GO/VEN/anti-OX40 antibody PF-04518600	PD-1 + CD33/HDACi/OX40 + HMA	AEs	NCT03390296
Ib	HMAs failure MDS	Pembrolizumab + entinostat	PD-1 + HDACi	MTD	NCT02936752
I	Untreated AML with TP53-mutated	Nivolumab + decitabine + venetoclax	PD-1 + HMA + HDACi	CRc	NCT04277442
II	previously untreated AML/sAML	Pembrolizumab + AZA + VEN	PD-1 + HMA + HDACi	MRD-CR	NCT04284787
I	Higher-risk MDS or AML with ≤ 30% blasts	Nivolumab + DAC + NY-ESO-1 vaccination^a^	PD-1 + HMA + tumor vaccine	Safety	NCT03358719
I/II	Higher-risk MDS/CML with HMA-failure	Atezolizumab + guadecitabine	PD-L1 + HMA	DLTs, ORR	NCT02935361
II	MDS with post injectable HMA-failure	Durvalumab and/or oral AZA	PD-L1 + HMA	ORR	NCT02281084
I	AML	Atezolizumab + Guadecitabine	PD-L1 + HMA	AEs, CR, CRp, CRi, DOR	NCT02892318
I	R/R AML	Atezolizumab + Hu5F9-G4	PD-L1 + CD47	AEs, CR, DOR	NCT03922477

*Allo-SCT* allogeneic stem cell transplantation, *AML* acute myeloid leukemia, *ALL* acute lymphoblastic leukemia, *CRi* complete remission with incomplete count recovery, *CRc* composite complete response, *DLTs* dose-limiting toxicities, *DOR* duration of response, *MTD* maximum tolerated dose, *RFS* recurrence-free survival, *SD* stable disease

^a^NY-ESO-1 vaccination: Anti-DEC-205-NY-ESO-1 fusion protein plus poly-ICLC

## Future perspectives

It is increasingly clear that PD-1/PD-L1 blocker-based treatment in MDS/AML faces overt challenges. Firstly, reliable biomarkers are necessary for predicting and tracking the responses of patients. Although a previous study has reported higher CD3^+^ T cells ratio in PB and BM samples from AML patients as a predictive biomarker [131], a large room for improvement remains. In solid tumors, predictive biomarkers of PD-1/PD-L1 blocker have been studied extensively. Soluble PD-L1 (sPD-L1), which is produced by DCs and cancer cell lines in *vitro*, has potential as a predictive biomarker [148]. Studies reported that elevated baseline sPD-L1 level is correlated with lower response rate of PD-1/PD-L1 inhibitors in non-small-cell lung cancer cases [149, 150]. DNA mismatch repair (MMR), which is vital for the maintenance of genomic stability, has been identified to play critical roles in the pathogenesis of AML [151]. Recent findings have suggested that a high degree of microsatellite instability can predict patient response to PD-1 blocker in colorectal cancer [152]. IFN-γ is secreted by activated CD8^+^ T cells to inhibit tumor cell proliferation, while it also upregulates PD-L1 on these cells [153]. Recent studies also have shown that melanoma patients with elevated baseline levels of IFN-γ exhibit a higher potential to respond to these agents [154, 155]. Therefore, the dynamic monitoring of PD-L1 expression during therapy can be used as an indirect assessment of the efficacy of PD-1/PD-L1 inhibitor, which exerts functions by restoring CD8^+^ T cell activation. Other biomarkers, including immunoscore, tumor mutational burden, gut microbiota and additional peripheral cytokines are being assessed eagerly, although their application values remain largely unclear in MDS/AML [156].

Moreover, peripheral blood cytopenia and decreased number of functional effector T cells are common signs of MDS/AML, abrogating the immune system, whereas pre-existing TILs are considered a critical factor for predicting a durable response to PD-1/PD-L1 blocker [157, 158]. Effective immune reconstitution and low disease burden following SCT may provide an ideal setting [159]. Therefore, PD-1/PD-L1 blockade following allo-HSCT is suggested, which may achieve a competent immune system to fully eradicate the underlying malignancy.

In addition to the aforementioned hypotheses, T cell regulation is an extremely complex process involving multiple inhibitory checkpoint molecules [160, 161]. Although PD-L1 expression is increased in MDS/AML blasts, and elevated PD-L1 expression on malignant cells is generally considered a reliable biomarker for predicting response to PD-1/PD-L1 blocker, a high number of MDS/AML patients do not fully benefit from these agents. Notably, recent evidence has suggested that PD-1/PD-L1 upregulation in MDS/AML patients less mediates the upfront tumor immune escape than reflects an adaptation resistance of tumor cells to the ongoing anticancer immunity, which is accompanied by PD-1 upregulation, co-expression of other immune checkpoints on effector T cells including TIM-3 [53, 137, 162], LAG-3 [75, 137], CTLA-4 [131, 163] and TIGIT [27].

**Fig. 4:**
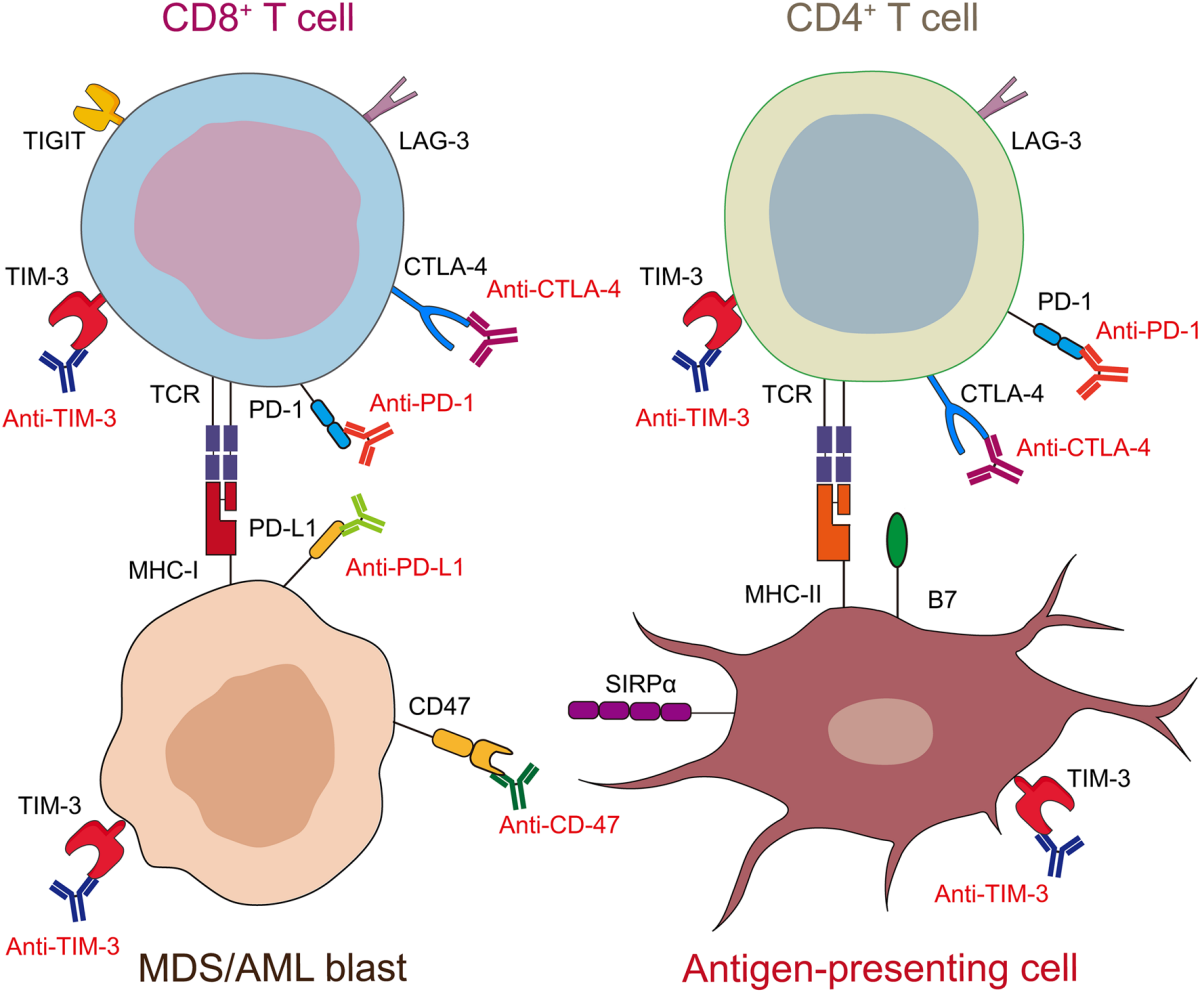
The prospect of novel immune checkpoint targets in MDS/AML treatment. An overview of the interactions between ICIs and immune checkpoints expressed on CD4^+^/CD8^+^ T cells, antigen-presenting cells and MDS/AML blasts in bone marrow of patients

Therefore, it may be difficult to achieve promising results by simply blocking PD-1/PD-L1 signaling alone. Combination of PD-1/PD-L1 inhibitor with other ICIs has been suggested [76, 164–167]. Other immune checkpoints (Fig. 4), e.g., CTLA-4 [68, 131, 163], TIM-3 [53, 162, 168–171], CD47 [172–174], LAG-3 [32, 75] and TIGIT [27, 30], have all been shown to be upregulated in MDS/AML patients. Several trials have assessed efficacy of these ICIs in treating MDS/AML, with promising preliminary results. The NCT02530463 trial assessed the efficacy of a double immune checkpoint inhibition by both CTLA-4 and PD-1 in the treatment of MDS. In the HMA-failure cohort with a 25-month median follow-up, the ORR was 36%, including 9% CR (1/11), 9% CRi (1/11) and 18% HI (2/11) [175]. In NCT03066648, the efficacy of a TIM-3 inhibitor in combination with DAC was assessed in 17 treatment-naïve HR-MDS and 38 AML patients. An encouraging result was reported with 50% MDS patients achieving mCR or CR, 14% and 14% newly diagnosed AML patients showing CR and PR, and 29% R/R AML patients achieving CRi [176]. Surprisingly, the anti-CD47 antibody magrolimab yielded encouraging outcome for the treatment of MDS/AML [177, 178]. In a phase Ib trial, 43 treatment-naïve patients (18 MDS and 25 AML) were treated with magrolimab in combination with AZA. In the MDS cohort, the ORR was 100%, with 54% cases achieving CR and 39% showing marrow CR. In the AML cohort, the ORR was 69%, with 50% cases showing CR or CRi. A good safety profile was also reported with most common AEs being anemia (37%), neutropenia (26%) and thrombocytopenia (26%). Only 1 patient developed treatment-related febrile neutropenia, and only 1 case had treatment discontinuation due to AEs [178]. Additional translational studies are needed to clarify roles of immune checkpoint blockade therapy in MDS/AML.

## Conclusion

Currently, novel treatment strategies beyond epigenetic drugs are highly required for the treatment of MDS/AML. The remarkable development of immune checkpoint therapy provides a novel therapeutic strategy. Inflammatory signaling pathways have been found to play central roles in leukemogenesis, which draws additional attention into the role of dysregulated immune checkpoints. Recent studies have found PD-1/PD-L1 are upregulated in MDS/AML patients and play vital roles in the pathogenesis of this disease. Further preclinical studies also reported the efficacy of PD-1/PD-L1 blocker in MDS/AML models and suggested a potent clinical efficacy. However, available clinical studies assessing PD-1/PD-L1 blockade have reported modest outcome improvement in MDS/AML patients. Future challenges include identifying reliable biomarkers, exploring more optimal combination therapies, and determining the subgroups of patients who might to benefit from PD-1/PD-L1 blocker. Furthermore, as other immune checkpoints (CTLA-4, TIM-3 and CD47) are also co-expressed with PD-1 in MDS/AML, future studies focusing on the interactions between different immune cells and immune checkpoint molecules in MDS/AML are warranted, and designing more reasonable dual or triplet combination therapies may also help.

## Data Availability

Data sharing is not applicable to this article as no datasets were generated or analyzed during the current study.

## Abbreviations

AEs: Adverse events
allo-HSCT: Allogeneic hematopoietic stem cell transplant
ALL: Acute lymphoblastic leukemia
AML: Acute myeloid leukemia
AZA: Azacitidine
CR: Complete response
CRp: CR with incomplete platelet recovery
CTLA-4: Cytotoxic T-lymphocyte-associated-protein 4
DAC: Decitabine
DLTs: Dose-limiting toxicities
MMR: Mismatch repair
ELN: European LeukemiaNet
FN: Febrile neutropenia
GVHD: Graft-versus-host disease
HD: Healthy donors
HDACi: Histone deacetylase inhibitors
HiDAC: High dose cytarabine
HMAs: Hypomethylating agents
HSPCs: Hematopoietic progenitor cells
HSCs: Hematopoietic stem cells
IDO1: Indoleamine 2,3-dioxygenase 1
IFN-γ: Interferon-γ
LICs: Leukemia-initiating cells
IL-6: Interleukin-6
IRAE: Immune-related adverse events
IPSS: International Prognostic Scoring System
IPSS-R: Revised International Prognostic Scoring System
ITD: Internal tandem duplications
LAG-3: Lymphocyte activation gene-3
MDS: Myelodysplastic syndromes
MDSCs: Myeloid-derived suppressor cells
MHC: Major histocompatibility complex
MT: Mutation
MTD: Maximum tolerated dose
NGS: Next-generation-sequencing
NPM1: Nucleophosmin
ORR: Overall response rate
OS: Overall survival
PBMNCs: Peripheral blood mononuclear cells
R/R: Relapsed/refractory
PD-1: Programmed cell death-1
PD-L1: Programmed cell death ligand-1
PR: Partial response
SAE: Serious adverse event
sAML: Secondary AML
SD: Stable disease
sPD-L1: Soluble PD-L1
STAT3: Signal transducer and activator of transcription 3
TCR: T-cell receptor
TIGHT: T cell immunoglobulin and ITIM domain
TIM-3: T-cell immunoglobulin mucin-3
TLS: Tumor lysis syndrome
TNF-α: Tumor necrosis factor-α
TNFR: TNF receptor
Tregs: Regulatory T cells
